# Hair Lead, Aluminum, and Other Toxic Metals in Normal-Weight and Obese Patients with Coronary Heart Disease

**DOI:** 10.3390/ijerph18158195

**Published:** 2021-08-03

**Authors:** Anatoly V. Skalny, Philippe Yu Kopylov, Monica M. B. Paoliello, Jung-Su Chang, Michael Aschner, Igor P. Bobrovnitsky, Jane C.-J. Chao, Jan Aaseth, Sergei N. Chebotarev, Alexey A. Tinkov

**Affiliations:** 1 World-Class Research Center “Digital Biodesign and Personalized Healthcare”, IM Sechenov First Moscow State Medical University (Sechenov University), 119435 Moscow, Russia; skalny3@microelements.ru (A.V.S.); fjk@inbox.ru (P.Y.K.); 2 Department of Bioelementology, KG Razumovsky Moscow State University of Technologies and Management, 109004 Moscow, Russia; rektorat@mgutm.ru; 3 Department of Molecular Pharmacology, Albert Einstein College of Medicine, New York, NY 10461, USA; monibas2@gmail.com (M.M.B.P.); michael.aschner@einsteinmed.org (M.A.); 4 Graduate Program in Public Health, Center of Health Sciences, State University of Londrina, Londrina 86057-970, Brazil; 5 College of Nutrition, Taipei Medical University, Taipei 110, Taiwan; susanchang@tmu.edu.tw (J.-S.C.); chenjui@tmu.edu.tw (J.C.-J.C.); 6 College of Public Health, Taipei Medical University, Taipei 110, Taiwan; jaol-aas@online.no; 7 Laboratory of Molecular Dietetics, IM Sechenov First Moscow State Medical University (Sechenov University), 119435 Moscow, Russia; 1ipb@mail.ru; 8 Centre for Strategic Planning of FMBA of Russia, 123182 Moscow, Russia; 9 Nutrition Research Center, Taipei Medical University Hospital, Taipei 110, Taiwan; 10 Research Department, Innlandet Hospital Trust, 2380 Brumunddal, Norway; 11 Laboratory of Ecobiomonitoring and Quality Control, Yaroslavl State University, 150003 Yaroslavl, Russia

**Keywords:** obesity, coronary heart disease, ischemic heart disease, lead, toxicity

## Abstract

The objective of the present study was to evaluate hair toxic metal levels in patients with obesity and/or coronary heart disease (CHD). Following a 2 × 2 factorial design, subjects without CHD were grouped into normal weight control (*n* = 123) and obese groups (*n* = 140). Patients suffering from CHD were divided into normal weight (*n* = 180) and obese CHD subjects (*n* = 240). Hair Al, As, Cd, Hg, Ni, and Pb levels were evaluated using inductively-coupled plasma mass-spectrometry. The data demonstrate that hair Al and Hg levels were higher in obese subjects as compared to normal weight controls. Normal weight CHD patients were characterized by significantly higher hair Al, As, Cd, and Pb levels when compared to healthy subjects. The highest hair Al, As, and Pb levels were observed in obese CHD patients, significantly exceeding the respective values in other groups. Factorial analysis revealed significant influence of factorial interaction (CHD*obesity) only for hair Pb content. Given the role of obesity as a risk factor for CHD, it is proposed that increased toxic metal accumulation in obesity may promote further development of cardiovascular diseases.

## 1. Introduction

Cardiovascular diseases (CVD) are considered as the leading cause of mortality accounting for 17.8 million deaths worldwide with more than 80% of global mortality observed in developing countries [1]. In turn, coronary heart disease (CHD) is associated with nearly one third of all deaths in adults older than 35 years [2].

The majority of CVD cases is associated with exposure to multiple risk factors, whereas only 2–7% of patients have no risk factors [3]. Dyslipidemia, smoking, diabetes, hypertension, sedentary lifestyle, unhealthy diet, stress, and obesity are considered as modifiable risk factors [4]. Results of meta-analysis involving 881,692 participants demonstrated that obesity is associated with 20% higher risk of CHD [5]. In turn, non-modifiable risk factors include age, sex, family history, and race [4].

In addition to other well-established factors, environmental pollution has also been shown to contribute significantly to increased risk of CHD [6]. Specifically, PM2.5 pollution was associated with nearly 1 million CHD deaths in 2017 [7]. Being in agreement with the existing data on toxic effects of cadmium [8], lead [9], arsenic [10], and aluminum [11] on the cardiovascular system, a detailed analysis demonstrated that exposure to metals including copper, cadmium, nickel, and palladium is associated with CHD rates [12]. In addition, recent studies demonstrated a significant association between increased titanium and arsenic body burden and CHD [13].

A significant impact of toxic metal exposure on obesity has been shown via the modulation of adipogenesis and adipose tissue functioning [14]. Correspondingly, cumulative metal exposure (lead, cadmium, mercury, arsenic, etc.) was found to be associated with obesity and associated diseases including hypertension and type 2 diabetes mellitus [15].

Despite significant attempts to decrease metal emissions into the environment, metal exposure is considered as significant health hazard. In parallel with occupational exposure to toxic metals, lifestyle factors including living in polluted environments, dietary and waterborne exposure, and smoking significantly contribute to increased accumulation of cadmium [16], lead [17], arsenic [18], and other metals in the organism, thus possessing significant adverse health effects.

Given the role of obesity as a risk factor for CHD, as well as the association between body metal accumulation with both obesity and CHD, it is reasonable to propose that toxic metal overexposure may potentiate the risk of these comorbidities. Therefore, the objective of the present study was to estimate whether comorbidity between obesity and CHD is associated with higher hair toxic metal accumulation.

## 2. Materials and Methods

### 2.1. Cohort Characteristics

The study was performed on a voluntary basis in agreement with the ethical principles of the Declaration of Helsinki and its later amendments (2013). All study subjects were informed about the objectives and details of experimental procedures and subsequently signed the informed consent form. The protocol was approved by the local ethics committee at Sechenov University, 07-17/13.09.17 (Moscow, Russia).

A total of 683 subjects living in Moscow for more than 3 years were enrolled in the current study. The study involved a 2 × 2 factorial design with grouping according to the presence of obesity and CHD. Based on BMI values, the participants were grouped into normal weight control group (*n* = 123) and obese group (*n* = 140) that were free of cardiovascular diseases. In turn, examinees suffering from CHD were divided into normal weight CHD patients (*n* = 180) and obese CHD subjects (*n* = 240) (Table 1). Power analysis demonstrated sufficient power of the study for assessment of direct effects of obesity and CHD at the used sample sizes.

The examined patients and controls were recruited at Center for Biotic Medicine (Moscow) at a voluntary basis. CHD was diagnosed according to the Clinical recommendations on coronary heart disease by Ministry of Health of the Russian Federation (2020). Data on diagnosis of CHD as well as other cardiovascular disease and comorbidities were extracted from the clinical records of the outpatient department. Only patients without acute coronary syndrome in anamnesis were involved in the current study.

Obesity was classified as BMI > 30. BMI was calculated using the values of body height (m) and weight (kg) according to the standard formula (BMI = body weight/height^2^). Anthropometric parameters were evaluated prior hair sampling.

In order to reduce the impact of side factors on the outcome of the study, the following exclusion criteria were used: current and former positive smoker status; use of synthetic chemical hair dyes; excessive alcohol consumption (more than 1 drink/day for women and 2 drinks/day for men); diabetes mellitus type 2; liver diseases including viral hepatitis and non-alcoholic fatty-liver disease (NAFLD); metal implants; occupational exposure to toxic metals and other toxicants (current and former); acute inflammatory diseases. Information on occupation and lifestyle factors were obtained during interviewing, whereas data on health status were extracted from outpatient medical records.

### 2.2. Hair Sampling and Preanalytical Treatment

On the day of sampling, the participants washed their hair with their day-to-day commercial shampoos. The proximal 1 cm of occipital hair strands (~0.1 g) were collected using ethanol-precleaned stainless steel scissors. The obtained hair samples were precleaned with acetone, rinsed thrice with distilled deionized water (18 MΩ cm) (Labconco Corp., Kansas City, MO, USA), and dried on air to a stable weight.

Washed hair samples were subjected to HNO_3_-assisted microwave digestion. Briefly, a total of 50 mg hair samples were introduced into Teflon tubes containing 5 mL of 65% HNO3 (Sigma-Aldrich Co., St. Louis, MO, USA). Microwave digestion was performed in the Berghof SpeedWave-4 DAP-40 (microwave frequency, 2.46 GHz; power, 1450 W) microwave system (Berghof Products + Instruments GmbH, 72800 Eningen, Germany) for 20 min with a maximal temperature of 170–180 °C.

After cooling the system, the digested samples were transferred into polypropylene tubes and adjusted with distilled deionized water (18 MΩ cm) to 15 mL.

### 2.3. Inductively-Coupled Plasma Mass-Spectrometry

Hair aluminum (Al), arsenic (As), cadmium (Cd), mercury (Hg), nickel (Ni), and lead (Pb) content were evaluated with inductively-coupled plasma mass-spectrometry at NexION 300D (PerkinElmer Inc., Shelton, CT, USA) equipped with the ESI SC-2 DX4 autosampler (Elemental Scientific Inc., Omaha, NE, USA). Each sample was assessed in triplicates.

The limits of detection (LoD) for Al, As, Cd, Hg, Ni, and Pb were 0.13, 0.001, 0.0016, 0.012, 0.009, and 0.0006 ppb, respectively. The values < LoD were obtained for Al, As, Cd, Hg, Ni, and Pb in 4, 25, 7, 19, 7, and 2 cases, respectively. Values < LoD were treated as zero results (0 µg/g), being out of 5–95 percentile range, and thus not affecting the matrix significantly.

Calibration of the ICP-MS system was performed using the standard metal solutions of 0.5–50 μg/L prepared from Universal Data Acquisition Standards Kits (PerkinElmer Inc., Shelton, CT, USA). Internal on-line standardization was performed using 10 μg/L yttrium-89 (^89^Y) and rhodium-103 (^103^Rh) standard solutions prepared from Yttrium (Y) and Rhodium (Rh) Pure Single-Element Standards (PerkinElmer Inc.).

The laboratory quality control procedure was performed daily using the certified reference materials of human hair GBW09101 (Shanghai Institute of Nuclear Research, Academia Sinica, China). The recovery rates for all studied metals were found within the limit of 87–112%.

### 2.4. Statistical Analysis

The obtained data were processed with Statistica 10.0 software (StatSoft, Tulsa, OK, USA). Data distribution was assessed using the Shapiro–Wilk test. Data characterized by skewed distribution were presented as Median and 25–75 percentile boundaries. To address the 2 × 2 design of the study, raw data were log transformed and further processed using two-way analysis of variance (ANOVA) with Fisher’s LSD post-hoc analysis for assessment of group difference significance. Factorial analysis was performed using two-way ANOVA to estimate the statistical influence of independent variables obesity (0/1) and CHD (0/1), as well as their interaction (obesity*CHD) on hair toxic metal content as a dependent variable. The correlation between hair metal content and anthropometric variables was evaluated with Spearman’s rank correlation coefficient. Multiple linear regression was also performed to reveal relative contribution of age, gender, BMI, and CHD status (0/1) on hair contents of each metal. All tests were considered significant at *p* < 0.05.

## 3. Results

The obtained data (Table 2) demonstrate that hair Al levels in obese subjects and CHD patients exceed the respective control values by 42% (*p* = 0.003) and 103% (*p* < 0.001). The highest hair Al content was observed in obese CHD patients, exceeding the levels in obese and CHD patients by 72% (*p* < 0.001) and 20% (*p* = 0.002), respectively. Hair Al level in obese CHD subjects was greater than 2.4-fold higher as compared to the control values (*p* < 0.001).

Hair As content did not differ significantly between normal weight and obese subjects (*p* = 0.120). In turn, patients with CHD were characterized by greater than twofold higher hair As content than in the control subjects (*p* < 0.001). Maximal hair As levels were also observed in obese CHD subjects, being greater than 3- and 2-fold higher than in normal weight and obese examinees (both *p* < 0.001). Moreover, hair As levels in obese CHD patients were 19% higher when compared to normal weight CHD subjects (*p* = 0.031).

Hair Cd content in normal weight CHD patients and obese CHD patients significantly exceeded the respective values in normal-weight and obese subjects without CHD by 157% and 83% (*p* < 0.001), respectively. At the same time, hair Cd levels in obese CHD subjects were more than threefold higher than those in control subjects (*p* < 0.001).

Analysis of hair Hg levels demonstrated no significant group difference between patients with CHD and examinees without cardiovascular diseases irrespective of body weight. Obese subjects and obese CHD patients were characterized by 30% (*p* = 0.025) and 21% (*p* = 0.034) higher hair Hg content as compared to normal-weight controls and lean CHD patients, respectively.

In contrast to other metals analyzed, no significant group difference was observed for hair Ni levels.

Hair Pb content in obese subjects was 41% higher when compared to normal-weight controls, although the difference was only nearly significant. At the same time, hair metal levels in normal-weight CHD patients significantly exceeded the respective control values by 15%. The highest hair Pb content was observed in obese CHD patients, being higher than that in control, obese, and normal-weight CHD examinees by 93%, 37%, and 68%, respectively.

Factorial analysis demonstrated a significant contribution of both obesity and CHD to hair metal levels (Table 3). Specifically, obesity had a significant effect on hair Al, As, Hg, and Pb content, and a nearly significant influence on hair Cd levels. Hair Al, As, Cd, and Pb were also significantly associated with the presence of CHD. At the same time, no significant influence of factorial interaction between obesity and CHD on hair metal levels was observed. Of the demographic covariates, gender had a significant effect on the levels of all analyzed metals in hair. In turn, age had a significant impact on hair Al, Cd, and Hg levels.

Given the noted impact of age and obesity on hair metal levels, correlation between height, weight, and BMI values was evaluated (Table 4). Hair Al, As, and Cd content was found to correlate inversely with subjects’ height, and was positively associated with body weight. Hair Pb content was characterized by a positive correlation with body weight and was unrelated to height.

Correlation analysis was also performed in order to reveal the association between toxic metal levels in hair of the examinees (Table 5). The obtained data demonstrate that hair Al levels positively correlated with As, Cd, Ni, and Pb content. Hair As concentration was found to be directly associated with hair Cd and Pb levels. Cd content in hair positively correlated with Ni and Pb levels, being inversely associated with hair Hg. Direct correlation between hair Pb and Ni concentration was also observed. At the same time, the revealed correlations were low, and only association between Cd and Pb levels was considered as moderate.

Multiple regression analysis evaluated the association between hair toxic metal levels as dependent variables and BMI and CHD status as independent variables after adjustment for age and sex (Table 6). BMI was found to be positively associated with hair Al and Pb levels, whereas CHD was considered as a significant predictor of increased hair Al, As, Cd, and Pb levels after adjustment for age and sex.

## 4. Discussion

Our novel data demonstrated a significant association between several toxic metals, CHD, and obesity. Increased levels of multiple toxic metals may be indicative of the importance of cumulative metal exposure in development and progression of these diseases.

Generally, toxic effects of cadmium [8], lead [9], arsenic [10], and aluminum [11] on the cardiovascular system may be associated with oxidative stress, inflammation, endothelial dysfunction, dyslipidemia, and atherogenesis, as well as direct cardiotoxicity. In turn, the role of toxic metal exposure in obesity may be associated with similar mechanisms, as well as the direct impact of metal toxicity on adipogenesis through modulation of key transcription factors including PPARγ and C/EBPα [14].

Being in agreement with the present findings, several studies also demonstrated an association between multiple toxic metal body burden and coronary heart disease and obesity. Specifically, serum Pb, Cd, and Hg levels were greater than 2-, 2-, and 1.5-fold higher in CHD patients compared to controls [19]. The present findings also corroborate results of a recent meta-analysis that demonstrated increased relative risk (RR) of 1.23, 1.85, and 1.29 for CHD in subjects with the highest tertile of As, Pb, and Cd levels as compared to the lowest tertile. Corroborating the present findings, Hg levels were not associated with CHD [20].

Correspondingly, cumulative metal exposure patterns including Pb, Cd, As, Ba, Hg, and Tl were also found to be associated with increased risk of obesity [15]. Our previous study demonstrated a significant BMI-associated increase in hair Cd, Hg, Pb, and Sn in adults [21]. However, in a recent meta-analysis of the existing data, we demonstrated the association between Pb and Hg, but not As and Cd, with metabolic syndrome [22].

At the same time, several additional studies, which aimed at evaluation of the interaction between single metal levels and CHD and/or obesity, demonstrated potential underlying mechanisms of cardiovascular toxicity.

Bone lead levels were directly associated with increased CHD risk after adjustment for lipid profile, BMI, smoking, and age. Further, the highly adipogenic Western diet was shown to increase the association between Pb and CHD [23]. It has also been demonstrated that the interaction between Pb exposure and CHD risk is modified by polymorphisms of the vitamin D receptor (VDR), heme oxygenase-1 (HMOX1), apolipoprotein E (APOE), angiotensinogen (AGT), and glutathione S-transferase (GSTP1) genes, indicating potential underlying mechanisms [24]. Additionally, it has been established that Pb-induced endothelial dysfunction can contribute to increased CVD risk [25]. In vivo and in vitro studies revealed the mechanisms of Pb-induced cardiovascular toxicity underlying increased risk of cardiac failure including Pb-induced oxidative stress, Ca2+ imbalance, inflammation, lipid metabolism dysregulation, alteration of RAAS and NO systems, etc. [26].

In the SPECT-China study, blood lead levels were found to be associated with increased odds for obesity [27]. Our earlier findings also demonstrated direct association between hair Pb content and BMI values in men and women [21]. Experimental data corroborated an effect of Pb on obesity in animal models [28]. These effects may include the effect of Pb on adipogenesis, as well as central regulation of food control and energy balance [14].

Cadmium exposure was also found to be associated with 1.34 higher hazard ratios of coronary heart disease in native Native American Indian adults [29]. Blood Cd levels upon environmental metal exposure were associated with a 10-ear risk of CHD in Koreans [30]. Heart failure was also associated with blood cadmium in a 17-year follow-up [31]. The results of a meta-analysis demonstrated that increased blood and urine Cd levels are associated with 59% and 34% higher CHD prevalence [32]. The underlying mechanisms of Cd-induced cardiovascular toxicity may involve oxidative stress, inflammation, endothelial dysfunction, dyslipidemia, and altered glycosaminoglycan synthesis [33].

Several studies have demonstrated the association between Cd exposure and obesity. Specifically, maternal Cd exposure was found to be associated with increased incidence of obesity in offspring, as demonstrated both in an epidemiological study and a zebrafish model [5]. It has been also demonstrated that obesity and Cd exposure may have significant interactive effects on the incidence of prediabetes [34]. At the same time, despite laboratory evidence of Cd-induced adipose tissue dysfunction, current data on the association between Cd exposure and obesity are insufficient [27].

Although data on the potential influence of Al on CHD are insufficient, a previous study demonstrated significantly higher plasma Al in CHD [13]. Furthermore, acute exposure and Al toxicosis affect the cardiovascular system [35].

In agreement with the present study, our previous observations demonstrated higher hair and urinary Al levels in obese subjects, being also associated with hypertension and non-alcoholic fatty liver disease [36]. These effects may be mediated by Al-induced mitochondrial dysfunction and increased ectopic lipid accumulation [37].

As is a known pollutant with a wide spectrum of toxic effects including cardiotoxicity. In 2015, dietary As exposure was associated with more than 4.3 million CHD cases [38]. Even low-level As exposure is associated with 28% higher risk of CHD [39]. The effects of As exposure on CVD and CHD in particular was shown to be dose-dependent [40]. A significant influence of AS3MT, NOS3, ICAM1, VCAM1, SOD2, and APOE gene polymorphisms on the interaction between As exposure and CHD was demonstrated [41]. The mechanisms of As-induced CVD may include oxidative stress, inflammation, and endothelial dysfunction, as well as lipid dyshomeostasis [42].

Despite consideration of As as a potential obesogen [43], existing epidemiological data are insufficient and contradictory. A recent study demonstrated that salivary As levels are directly associated with BMI in women [44]. In contrast, urinary As concentration was characterized by inverse association with BMI in Taiwanese subjects [45].

Hair Hg was found to be associated only with obesity, but not CHD, in agreement with results of the previous meta-analysis [20]. In turn, certain studies demonstrated the association between Hg exposure and obesity [46]. Blood Hg levels were also associated with visceral adipose tissue volume in healthy adults [47]. However, certain contradictions in the field exist as well. Specifically, blood Hg levels were shown to be directly associated with obesity in Koreans [48], yet inversely correlated with BMI in American adults [49]. Our previous study also revealed positive association between high hair Hg and BMI as well as age in men and women [21], and these related to an adverse metabolic profile [50]. Experimental studies demonstrated that adipose tissue could be considered as a target tissue for Hg toxicity subjected to significant morphologic alterations [51]. The latter may be mediated by the interference of Hg with key adipogenic transcription factors [14].

Although experimental data on the impact of metals on obesity and CHD exist, the potential impact of obesity and CHD on increased body metal accumulation is also possible. One of the mechanisms of metal detoxification involves metallothionein expression that binds toxic metals, thus limiting its hazardous effects. At the same time, it has been demonstrated that CHD is associated with MT2A gene polymorphisms and misfunction [52]. In turn, increased MT expression possesses cardioprotective effects [53]. Correspondingly, experimental MT deficiency is also associated with altered energy metabolism resulting in increased lipid accumulation in adipose tissue [54] and obesity [55]. Hypothetically, altered metallothionein expression patterns in obesity and/or CHD may underlie the observed increase in toxic metal accumulation, which may characterize an inverse causal relationship between the studied diseases and toxic metal levels.

Despite the large sample size, the present study has certain limitations. Specifically, cross-sectional design of the study does not allow us to estimate the causal relations between toxic metal accumulation and obesity and/or CHD. In turn, follow-up studies would be highly beneficial to evaluate the potential contribution of metal accumulation into the incidence of obesity and CHD. Although hair is considered as a long-term indicator of body metal burden, being indicative of previous 1–2 months exposure, this scale is insufficient to evaluate body metal levels prior to CHD and obesity manifestation. Therefore, the main limitation of the present study is lack of the estimated causal relations between metal exposure and cardiometabolic diseases.

## 5. Conclusions

Taken together, the present findings combined with results from previous observations support an association between toxic metal exposure with obesity and CHD.

Specifically, increased Al, As, and Pb body burdens were shown to be associated with both obesity and CHD. In turn, Hg and Cd were found to be associated only with obesity and CHD, respectively. It is notable that only high hair Pb levels were found to be associated with factorial interaction between obesity and CHD, being indicative of the potential contribution of this metal to comorbidity between obesity and CHD.

Although the cross-sectional design of the study does not highlight any causal relationship between metal exposure and metabolic diseases, clear toxicological evidence demonstrates that increasing body burdens of these metals may significantly aggravate metabolic disturbances, thus leading to disease progression.

Given the role of obesity as a risk factor of CVD and CHD in particular, it is proposed that increased toxic metal accumulation in obesity may promote further development of CVD. It is proposed that reduction of toxic metal exposure may significantly contribute to reduced metabolic risk in obesity and decreased incidence of CHD. At the same time, further epidemiological and laboratory studies are required in order to evaluate the contribution of toxic metal exposure on the risk and interactive effects of obesity and CHD.

## Figures and Tables

**Table 1:** Demographic and anthropometric parameters of the examinees.

Parameter	Lean Control (*n* = 123)	Obese (*n* = 140)	Lean CHD (*n* = 180)	Obese CHD (*n* = 240)
Sex, *n* (F/M)	101/22	103/37	126/64	166/74
Age, y.o.	51.1 ± 9.7	53.3 ± 7.2	55 ± 9.6	52.2 ± 8.6
Height, cm	167.5 ± 7.1	167.4 ± 8.9	165.2 ± 9.2	165.1 ± 8.7
Weight, kg	64.2 ± 6.9	94.2 ± 12.4	62.8 ± 8.2	94.4 ± 17.8
BMI	22.8 ± 1.4	33.6 ± 2.9	22.9 ± 1.7	34.5 ± 5.2

Data are expressed as means and the respective standard deviations; F—female; M—male.

**Table 2:** Hair toxic metal content (µg/g) in normal-weight and obese patients with coronary heart disease and healthy controls.

Metal	Lean Control (*n* = 123)	Obese (*n* = 140)	Lean CHD (*n* = 180)	Obese CHD (*n* = 240)
Al	2.864 (2.053–5.101)	4.056 ^1^ (2.784–6.803)	5.810 ^1,2^ (3.57–8.595)	6.971 ^1,2,3^ (4.321–11.085)
As	0.012 (0.006–0.023)	0.016 (0.008–0.026)	0.032 ^1^ (0.018–0.058)	0.038 ^1,2,3^ (0.021–0.068)
Cd	0.007 (0.005–0.017)	0.012 (0.007–0.021)	0.018 ^1^ (0.01–0.041)	0.022 ^1,2^ (0.011–0.051)
Hg	0.411 (0.232–0.701)	0.535 ^1^ (0.308–0.863)	0.425 ^2^ (0.238–0.642)	0.513 ^3^ (0.257–0.850)
Ni	0.232 (0.138–0.36)	0.188 (0.127–0.314)	0.194 (0.13–0.379)	0.231 (0.135–0.368)
Pb	0.276 (0.141–0.546)	0.390 (0.169–0.851)	0.317 ^1^ (0.177–0.595)	0.534 ^1,2,3^ (0.237–1.075)

Data are expressed as medians and the respective IQR; ^1,2,3^—significant difference in comparison to the control, obese, and normal weight CHD groups at *p* < 0.05, respectively.

**Table 3:** Factorial analysis of the association between the presence of obesity, coronary heart disease, and their interaction with hair toxic metal content adjusted for age and gender.

Factor	Al	As	Cd	Hg	Ni	Pb
Obesity	<0.001 *	0.020 *	0.074	0.003 *	0.667	<0.001 *
CHD	<0.001 *	<0.001 *	<0.001 *	0.644	0.279	0.024 *
Obesity * CHD	0.543	0.426	0.524	0.553	0.269	0.301 *
Gender	0.025 *	<0.001 *	<0.001 *	<0.001 *	<0.001 *	<0.001 *
Age	0.037 *	0.760	0.005 *	0.002 *	0.190	0.569

Data are expressed as *p* values for factorial influence as followed from two-way ANOVA; *—significance at *p* < 0.05.

**Table 4:** Correlation analysis of the association between hair toxic metal levels and anthropometric parameters in the studied cohort.

Metal	Height		Weight		BMI	
	r	*p*	r	*p*	r	*p*
Al	−0.295	<0.001 *	0.368	<0.001 *	0.159	<0.001 *
As	−0.397	<0.001 *	0.501	<0.001 *	0.086	0.027 *
Cd	−0.297	<0.001 *	0.359	<0.001 *	0.053	0.171
Hg	0.057	0.139	0.004	0.918	0.060	0.120
Ni	−0.016	0.686	0.047	0.225	-0.002	0.954
Pb	−0.044	0.253	0.186	<0.001 *	0.145	<0.001 *

Data are expressed as correlation coefficient (r) and the respective *p* values; *—correlation is significant at *p* < 0.05.

**Table 5:** Correlation between hair metal levels in the studied cohort.

p/r	Al	As	Cd	Hg	Ni	Pb
Al	-	0.409	0.421	−0.008	0.228	0.359
As	<0.001 *	-	0.421	0.055	0.047	0.336
Cd	<0.001 *	<0.001 *	-	−0.095	0.347	0.563
Hg	0.830	0.164	0.015 *	-	0.051	0.033
Ni	<0.001 *	0.234	<0.001 *	0.192	-	0.297
Pb	<0.001 *	<0.001 *	<0.001 *	0.404	<0.001 *	-

Data are expressed as correlation coefficient (r) (upper right part) and the respective *p* values (lower left part); *—correlation is significant at *p* < 0.05.

**Table 6:** Multiple linear regression analysis of the association between hair toxic metal levels (dependent variables) and BMI and CHD status (independent variables) after adjustment for age and sex of the examinees.

Metal	Al		As		Cd		Hg		Ni		Pb	
Predictor	β	*p*	β	*p*	β	*p*	β	*p*	β	*p*	β	*p*
BMI	0.133	<0.001 *	0.058	0.076	0.034	0.034	0.063	0.097	−0.003	0.927	0.142	<0.001 *
CHD	0.382	<0.001 *	0.440	<0.001 *	0.259	0.259	0.024	0.586	0.060	0.186	0.098	0.021*
Sex	0.085	0.017 *	0.295	<0.001 *	0.201	0.201	0.169	<0.001 *	0.147	<0.001 *	0.307	<0.001 *
Age	−0.093	0.026 *	0.003	0.933	0.113	0.113	−0.147	0.001*	−0.069	0.124	−0.031	0.455
Multiple R	0.396		0.564		0.406		0.225		0.165		0.359	
Multiple R^2^	0.157		0.318		0.165		0.051		0.027		0.129	
Adjusted R^2^	0.152		0.314		0.160		0.045		0.021		0.124	
P model	<0.001 *		<0.001 *		<0.001 *		<0.001 *		0.001 *		<0.001 *	

Data are expressed as regression coefficients (β) and the respective *p* values; *—association is significant at *p* < 0.5.

## Data Availability

Data available on request.
